# Abnormal Congenital Location of Stapes' Superstructure: Clinical and Embryological Implications

**DOI:** 10.1155/2016/2598962

**Published:** 2016-08-28

**Authors:** Vânia Henriques, Rafaela Teles, Ana Sousa, Roberto Estevão, Jorge Rodrigues, Alexandra Gomes, Francisco Silva, Ângelo Fernandes, Fausto Fernandes

**Affiliations:** Otorhinolaryngology Department, Hospital de Guimarães, Rua dos Cutileiros, 4835-044 Guimarães, Portugal

## Abstract

Congenital middle ear malformations are rare. Most part of them are usually associated with other malformations, such as aural atresia, microtia, and dysmorphic craniofacial features. A clinical case of a 24-year-old male with a right-sided conductive hearing loss since his childhood, without craniofacial malformation, is presented. He was proposed for exploratory tympanotomy under the suspicious diagnosis of otosclerosis. The surgery revealed an abnormal location of stapes' superstructure, which was attached to the promontory and had an isolated and mobile osseous footplate in the oval window. A stapes prosthesis was inserted and resulted in closure of the air-bone gap by 25 dB. A review of the literature was also performed using MEDLINE. Two theories diverge on the embryologic origin of the stapes. Our findings seem to be in favour of the theory that defines two different embryologic origins to the stapes.

## 1. Introduction

Congenital ossicular chain malformations are present in less than 1 per 15.000 births [1]. Ossicular anomalies include absence or malformation of any ossicles and may be framed into major and minor categories. Major abnormalities are associated with other malformations, such as aural atresia, microtia, and dysmorphic craniofacial features, thus involving the external auditory canal (EAC) and the pinna as well as the tympanic cavity and middle ear ossicles.

Minor abnormalities appear with a normal tympanic membrane, EAC, and pinna and may involve fixations, defects, or absence of middle ear structures (malleus, incus, stapes, oval window, and round window). These minor middle ear abnormalities can appear to be isolated or in combination, involving more than one structure [2, 3].

In middle ear abnormalities, patients classically present a nonprogressive conductive hearing loss and their most frequent problems are related to stapes ankylosis, discontinuity of the incudostapedial complex, or congenital absence of the stapes [4]. Ossicular chain embryological development is under investigation for nearly two centuries and two main theories, regarding stapes origin, have been proposed. The first hypothesizes that stapes has a dual origin source and the second postulates a single origin [5–7].

This is the report of a clinical case that may contribute to our knowledge of the stapes development.

## 2. Material and Methods

A 24-year-old healthy male was referred to Ear Nose and Throat consultation by long standing right ear hearing loss. He had no history of middle ear infection, neither of head injury, or even family history of hearing loss. In physical examination, he had no dysmorphic craniofacial features, presenting a normal pinna, EAC, and tympanic membrane. The otoscopy did not reveal any alteration; Rinne test was negative on the right side and neurological examination was normal.

**Figure 1:**
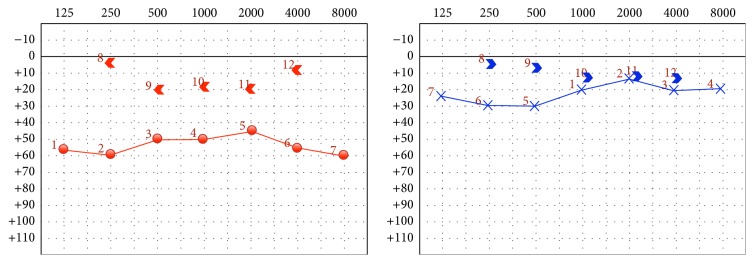
Right ear in red and left ear in blue.

Pure tone audiometry proved a moderate conductive hearing loss in the right ear, with a 4-frequency (500, 1000, 2000, and 4000 Hz) mean of 33 dB, and borderline normal thresholds in the left ear (Figure 1). Tympanometry revealed a normal ear canal volume and a normal tympanic membrane compliance. The acoustic reflexes were absent in right ipsilateral and left contralateral sides.

**Figure 2:**
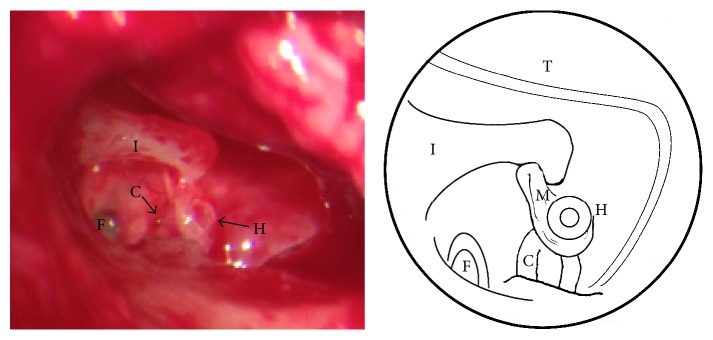
Intraoperative view of the middle ear (photo and diagram). After performing tympanotomy a disconnection between stapes superstructure and incus with a normal stapes footplate is observed. I: long process of the incus; H: head of the stapes; C: stapes crura; F: stapes footplate; T: tympanomeatal flap; M: mucosal folds.

The possibility of being an otosclerosis was pondered and so an exploratory tympanotomy was performed. During the procedure a congenital stapes abnormality was detected. The stapes' superstructure was well formed with two limbs and head, inferiorly located, separated from the footplate, and attached to the promontory, as seen in Figure 2. The footplate was normally located in the oval window's niche, was mobile, and had an osseous consistency. There was no viable incudostapedial joint, only the presence of mucosal folds, suspended between lenticular process of the incus and the head of the stapes. Besides this, the stapedius muscle tendon was not formed, despite pyramidal eminence being present. Malleus and incus were normally formed and in their anatomic position. There was no sign of congenital cholesteatoma after inspection of all middle ear cavity.

**Figure 3:**
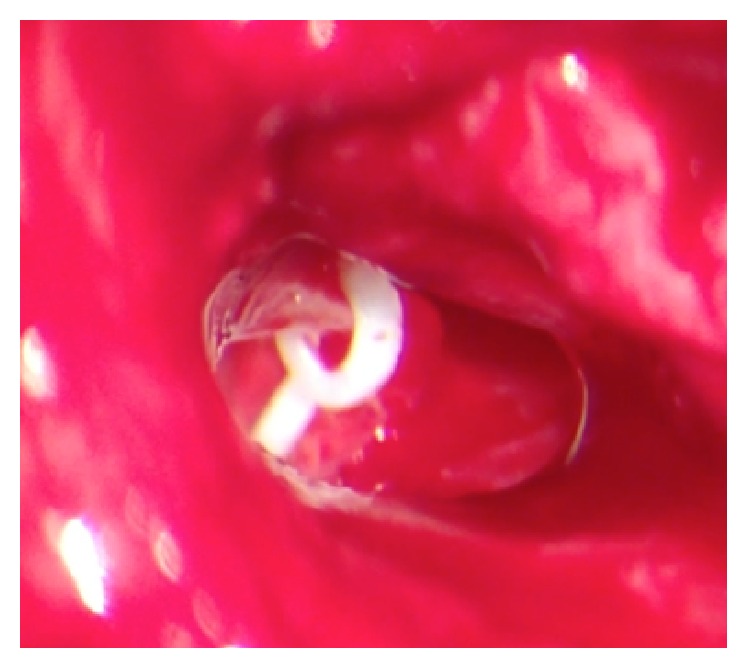
Placement of a fluoroplastic piston prosthesis.

The stapes' superstructure was removed from the promontory and then a stapedotomy was performed, creating a small fenestra in the centre of the footplate, in spite of being a mobile footplate. A fluoroplastic piston prosthesis was carefully placed (see Figure 3) and the fenestra was sealed with Gelfoam®.

**Figure 4:**
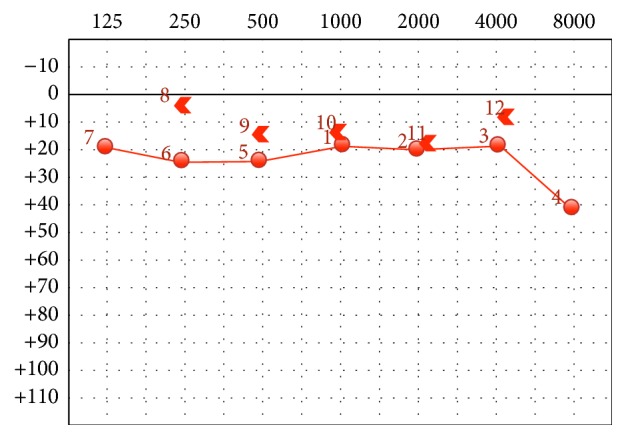
Postoperative audiogram.

The postoperative audiogram, Figure 4, revealed auditory restoration with the preoperative air-bone gap of 33 dB turning into 6 dB. No vertigo was reported postoperatively.

## 3. Discussion

The ossicular chain is formed by three ossicles, from lateral to medial: the malleus, the incus, and the stapes. Mechanical vibrations are transmitted into the inner ear structures via the integration of the stapes footplate and annular ligament into the oval window. Defects in the insertion of the stapes in the oval window produce a conductive hearing loss. A nonprogressive conductive hearing loss, in the range of 40 to 60 dB, with a normal tympanic membrane and no history of trauma or infection, is highly suggestive of a congenital ossicular malformation. As the preoperative diagnosis of congenital conductive hearing loss is challenging, an exploratory tympanotomy is an effective diagnostic method.

The development of the middle ear's ossicles, in humans, is not yet totally understood. This way, there are conflicting opinions regarding the stapes origin, namely, about the possible contribution of the otic capsule to the stapedial footplate. Two main theories have been proposed to demonstrate the stapes origin. The first theory, described in 1942 by Cauldwell and Anson and later confirmed by Masuda et al. (1978), hypothesizes that the stapes has a dual source. The superstructure and the tympanic part of the footplate develop from Reichert's cartilage (second branchial arch) and the vestibular part of the footplate develops from the otic capsule, thus both parts developing independently and later merging [5–7].

On the other hand, several authors suggest that Reichert's cartilage is the common and unique origin of these structures. In 2003 Louryan et al. presented a case report of a unilateral congenital stapes misplacement, revealed by CT, without any relationship with the otic capsule; hence they assumed that the otic capsule is not mandatory for stapes development [8]. However, later in 2005, Rodríguez-Vázquez published an article where he proposed the stapes development from the cranial mesenchyme of the second arch, separately from the Reichert's cartilage and independently from the optic capsule [9]. In the same year, O'Gorman suggested, based on lineage studies in mice, a mixed origin for the stapes [10]. Additionally, in 2012, Thompson et al. provided more evidence in this long standing controversy, using transgenic mice and showing that the oval window and stapes' footplate share the same embryonic origin [11]. Data from human development, using CT scans or following exploratory tympanotomy, advocate that, in the majority of cases when the oval window is absent, the stapedial footplate is absent, reduced, or displaced. According to Jahrsdoerfer, in a study of 51 ears with congenital absence of the oval window, a normal stapes' footplate was only observed in 4% of patients, confirming the important relationship between these two structures [4, 12].

In the present clinical case, a complete isolated stapes' superstructure misplacement was observed in the promontory, while having a mobile osseous footplate in the oval window. Stapes' crura were attached to the promontory by a thin bony layer. These findings may suggest a dual origin of the stapes, as supported by Cauldwell and Anson theory, of a dual origin to the stapes' footplate. Whittmore, in 2013, and later Kanona, in 2015, described similar cases of stapes' superstructure misplaced inferiorly and with a mobile footplate in the absence of trauma [13, 14]. Nevertheless, we cannot reject the hypothesis of these findings to be related to a unique anlage which, somehow, suffered a misdevelopment.

The Teunissen and Cremers classification of congenital anomalies of the middle ear is the most generally accepted (Table 1). This classification system has four main categories and is based on the different surgical reconstructive techniques available for the diverse minor anomalies. The present clinical case belongs to the “3(A) class.” This class is characterized by anomalies in malleus, incus, or stapes' superstructure, which cause a discontinuity in the ossicular chain, although with a normal and mobile footplate. According to Teunissen and Cremers, these anomalies are variations of developmental disorders and reconstruction of the ossicular chain should be performed whenever possible [15]. It is generally accepted that in patients with normal stapes footplate, stapedotomy with prosthesis insertion is the best surgical option. Even so, nonsurgical intervention should always be considered, once the management of congenital ossicular abnormalities depends on the type of ossicular malformation, technical issues, and patient factors.

## 4. Conclusion

Congenital minor abnormalities of the middle ear are rare causes of nonprogressive conductive hearing loss. Discontinuity between the stapes' superstructure and footplate was observed in this case report. The stapes' superstructure was attached to the promontory by a thin bony layer and totally disconnected from a thin footplate. There was no incudostapedial joint, only the presence of mucosal folds suspending between lenticular process of the incus and the head of the stapes. The footplate was mobile in the oval window. These findings seem to be in favour of the theory that defines two different embryologic origins to the stapes. Nevertheless, this is an area under actual investigation and further developments are expected.

## Figures and Tables

**Table 1:** Classification of congenital anomalies of the middle ear. Adapted from Teunissen and Cremers [15].

Class	Main anomaly	Subclassification
1	Congenital stapes ankylosis	

2	Stapes ankylosis associated with another congenital ossicular chain anomaly	

3	Congenital anomaly of the ossicular chain but mobile stapes footplate	(A) Discontinuity in ossicular chain (B) Epitympanic fixation

4	Congenital aplasia or severe dysplasia of oval window or round window	(A) Aplasia (B) Dysplasia (i) Crossing facial nerve (ii) Persistent stapedial artery
